# Feasibility and efficacy of nasal rehabilitation on nasal symptoms in patients with chronic allergic rhinitis: A pilot study

**DOI:** 10.1016/j.jacig.2026.100674

**Published:** 2026-02-17

**Authors:** Sachin Tendulkar, Prem Venkatesan, Satyanarayana Mysore, Vani Lakshmi R

**Affiliations:** a Department of Physiotherapy, Manipal College of Health Professions, Bengaluru, Manipal Academy of Higher Education, Manipal, Karnataka, India; b Department of Pulmonology, Manipal Hospitals, Bengaluru, Karnataka, India; c Department of Data Science, Prasanna School of Public Health, Manipal Academy of Higher Education, Manipal, Karnataka, India

**Keywords:** Chronic allergic rhinitis, nasal rehabilitation, nasal symptoms, mouth breathing

## Abstract

**Background:**

Chronic allergic rhinitis (CAR) is a highly prevalent condition characterized by nasal symptoms and mouth breathing. The detrimental effects of frequent pharmacologic treatment necessitate a nonpharmacologic treatment approach for patients with CAR.

**Objective:**

We sought to assess the feasibility, acceptability, safety, and preliminary effects of a nasal rehabilitation program in patients with CAR.

**Methods:**

A pilot study was conducted with 35 patients who underwent a nasal rehabilitation program for 5 weeks. The feasibility of the intervention from patient and therapist perspectives, adherence to the treatment, and occurrence of adverse events was recorded posttreatment. The preliminary effects of the treatment on nasal and mouth-breathing symptoms and disease-specific quality of life were assessed at baseline and after 5 weeks of treatment.

**Results:**

An adherence of 92.5% was observed for the treatment. The nasal rehabilitation techniques were feasible for patients and the therapist without any adverse events. The intervention demonstrated significant preliminary effects (*P* < .05) on the Total Nasal Symptom Score, the Nasal Obstruction Symptom Evaluation, and the Rhinoconjunctivitis Quality of Life Questionnaire, with a mean improvement of 2.00, 1.06, and 0.94 points, respectively.

**Conclusions:**

Nasal rehabilitation intervention is feasible, acceptable, and safe in patients with CAR. The treatment showed positive preliminary effects with reduced nasal symptoms and enhanced disease-specific quality of life among the patients.

The Allergic Rhinitis and its Impact on Asthma defines chronic allergic rhinitis (CAR) as “a persistent allergic rhinitis in which the symptoms occur at least 4 days a week over a period of at least 4 weeks.”^1^ The global prevalence of allergic rhinitis among adults is more than 400 million.^2^ Its prevalence in India is approximately 20% to 30%.^3^ Allergic rhinitis constitutes about 55% of all existing allergies and is among the top 10 reasons leading to frequent visits to primary care physicians.^3^ One of the major risk factors for increase in prevalence is global urbanization.^4^^,^^5^

CAR is associated with high prevalence and severity of asthma and results in worsened symptoms among individuals with chronic sinusitis and asthma.^6^^,^^7^ The deleterious effects of CAR include postnasal drip, persistent nasal congestion, mouth breathing, and disturbed sleep leading to daytime fatigue.^3^ Among these, mouth breathing raises serious concerns such as reduction in oral hydration, decreased mucociliary clearance, decreased local innate immune defense, and mucosal homeostasis. Furthermore, it results in reduction of intraoral space causing obstruction of the pharyngeal airway and nasal muscle dysfunction.^8^, ^9^, ^10^, ^11^, ^12^

Pharmacologic treatment of CAR consists of intranasal corticosteroids, antihistamines, decongestants, leukotriene receptor antagonists, and immunotherapy. The new-generation antihistamines are less likely to cause undesirable side effects.^13^ However, chronic nasal decongestant use leads to dryness, anxiety, and headache.^14^ In addition, chronic stimulation of α-adrenergic receptors induces receptor downregulation, contributing to decreased vasoconstriction and rebound congestion. This directs the patient to increase dosage, further increasing dependency on these drugs.^15^ Surgical approaches in allergic rhinitis include inferior turbinate reduction, outfracture lateralization, laser vaporization, radiofrequency ablation and coablation, submucosal resection, septoplasty, and endoscopic sinus surgery.^16^ The surgery improves nasal patency; however, it fails to control the inflammatory process and mouth-breathing pattern in patients with CAR.^17^^,^^18^

The detrimental effects of frequent pharmacologic treatment necessitate a nonpharmacologic treatment approach for patients with CAR. A study by Nair et al^19^ revealed that 3 months of humming intervention significantly reduced nasal symptoms in patients with allergic rhinitis. Furthermore, Courtney et al^20^ conducted a study on individuals with self-reported mouth breathing who underwent 4 weeks of online functional nasal rehabilitation. The study showed significant improvement in self-perceived nasal obstruction and subjective mouth breathing during day and night among the participants.^20^ However, there is a paucity of literature in addressing mouth breathing and nasal symptoms in patients with CAR. The aim of the present study was to assess the feasibility, acceptability, safety, and preliminary effects of a nasal rehabilitation program in patients with CAR.

## Methods

The present study is a pre/post pilot trial conducted in the Physiotherapy Department of Manipal Hospitals, Bengaluru, following approval from the hospital’s ethics committee. A total of 35 eligible individuals participated in the study. An informed consent was obtained from all the participants. The CONSORT extension of feasibility and pilot trials was followed for reporting the study.

### Inclusion criteria

Participants included in the study were males and females aged between 18 and 40 years diagnosed with CAR (duration, >4 weeks) by a pulmonologist on the basis of the Allergic Rhinitis and its Impact on Asthma guidelines.^21^

### Exclusion criteria

Participants were excluded if they were diagnosed with acute seasonal allergic rhinitis or had a history of craniofacial syndromes such as Down syndrome, Treacher Collins syndrome, Crouzon syndrome, or Apert syndrome. Individuals with a history of tracheostomy or laryngeal, subglottic, or pulmonary airway stenosis or those who had undergone surgeries, had presence of severe psychological disorders, had other lower respiratory tract conditions, or had a history of smoking were excluded.

### Procedure

An informed consent was obtained from the participants and then they underwent a structured nasal rehabilitation program for 5 weeks. The treatment protocol was obtained from a study by Courtney et al^20^ The participants were instructed to perform the prescribed exercises twice daily, with tele-rehabilitation sessions conducted by the physiotherapist at the end of each week to ensure adherence and proper technique. The adherence was assessed through an exercise diary recording the number of sessions performed by the patients daily. The intervention was administered by a qualified physiotherapist. Outcome measures were recorded at baseline and after the completion of the 5-week intervention. The nasal rehabilitation program comprised 3 components: humming and its variations, breath-holding techniques, and nose-opening smile (for details, see this article’s Methods section in the Online Repository at www.jaci-global.org). All the patients were on intranasal corticosteroids, namely, fluticasone furoate. The participants also underwent counseling regarding allergy avoidance, importance of medications, and their adherence along with the nasal irrigation technique, which was performed before the administration of intranasal corticosteriods.

### Outcomes

Feasibility of the intervention was evaluated using a 5-point Likert scale (0 = strongly disagree to 4 = strongly agree) from both patient and therapist perspectives. Patients rated the following statements: (1) the intervention was easy to understand, (2) they felt comfortable participating, (3) the instructions were clear, and (4) they would be willing to continue the intervention in the future. Therapist-reported feasibility included ratings on (1) ease of delivering the intervention, (2) patient comprehension and adherence, and (3) compatibility of the intervention with routine clinical practice. This dual-perspective evaluation was conducted to determine the acceptability, clarity, and clinical integration of the intervention.

The Total Nasal Symptom Score (TNSS) was used to assess the severity of nasal symptoms, comprising the sum of 4 individual symptom scores: rhinorrhea, sneezing, nasal congestion, and nasal itching. Each symptom was rated on a 4-point scale ranging from 0 to 3, where 0 indicated no symptoms, 1 indicated mild, easily tolerated symptoms, 2 indicated bothersome but manageable symptoms, and 3 indicated severe symptoms that were difficult to tolerate and interfered with daily activities, yielding a total score ranging from 0 to 12. The Cronbach α for TNSS is 0.87 with an Intraclass Correlation Coefficient (ICC) of 0.87.^22^

The Rhinoconjunctivitis Quality of Life Questionnaire (RQLQ) was used to evaluate disease-related quality of life and includes 36 items across 6 domains: nasal symptoms, ocular symptoms, activity limitations related to school or work, sleep disturbances, social functioning, and emotional well-being. In addition, 2 independent items assessed overall health and the number of days missed from work or school because of allergic rhinitis. The Cronbach α for RQLQ is 0.92 with an ICC of 0.92.^23^

The Nasal Obstruction Symptom Evaluation (NOSE) scale was used to quantify the impact of nasal obstruction on quality of life. It consists of 5 items rated on a 5-point Likert scale (0 = no problem to 4 = severe problem), with total scores calculated by summing item scores and multiplying the total by 5, yielding a final score ranging from 0 (no obstruction) to 100 (most severe obstruction). These validated outcome measures were selected to comprehensively capture symptom burden and functional impairment in patients with allergic rhinitis. The Cronbach α for NOSE is 0.88 with an ICC of 0.803.^24^

The internationally validated English versions of the outcomes were used for the study. All the outcome assessors were blinded to the study.

### Statistical analysis

Descriptive statistics were used to summarize the data, with means and SDs reported for continuous variables and frequencies and percentages reported for categorical variables. The normality of the data distribution was assessed using the Shapiro-Wilk test. Pre- and postintervention comparisons were performed using the paired *t* test for normally distributed data and the Wilcoxon signed-rank test for nonnormally distributed data. All statistical analyses were conducted using Jamovi software (version 2.3.28, Sydney, Australia), and a *P* value less than .05 was considered statistically significant.

## Results

All 35 participants enrolled in the study completed the intervention, with no dropouts. The baseline characteristics of the participants are provided in Table I. All 35 patients had perennial and chronic allergic rhinitis, which used to worsen seasonally.

**Table I:** Baseline characteristics of study participants (n = 35)

Characteristics	Mean ± SD/n
Age (y)	32.71 ± 6.311
Sex, n	
Male	19
Female	16
TNSS	1.971 ± 2.514
NOSE	41.286 ± 23.211
RQLQ	1.292 ± 0.751

The average adherence for the treatment session was 92.5%. The patient responses toward feasibility questions were as follows: For the first, “The intervention was easy to understand,” 15 participants rated it as 4 (strongly agree) and 20 participants rated it as 3 (agree). For the next, “I felt comfortable participating in the intervention,” all 35 participants gave a rating of 4 (strongly agree). For the third, “The interventions were easy to perform,” 30 participants rated it as 3 (agree) and 5 participants rated it as 4 (strongly agree). For the fourth question, “They would be willing to continue the intervention in the future,” 27 participants rated 4 (strongly agree) and 8 participants rated 3 (agree). From the therapist’s perspective, for all 3 statements, namely, “The intervention was easy to deliver,” “Patients were able to understand and follow the intervention,” and “The intervention was compatible with existing clinical practices,” the therapist had given a rating of 4 (strongly agree).

**Fig 1:**
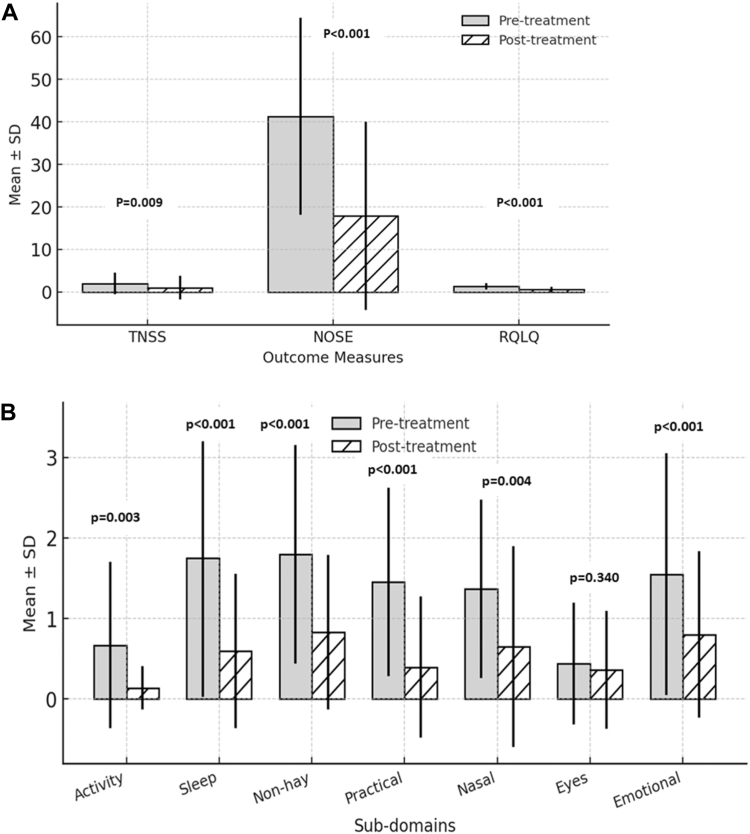
Effect of 5 weeks of nasal rehabilitation treatment. **A,** Amount of change (mean ± SD) in TNSS, NOSE, and RQLQ outcomes. **B,** Amount of change (mean ± SD) in subdomains of RQLQ outcome.

Following the intervention, statistically significant improvements were observed across all outcome measures. The mean differences for TNSS, NOSE, and RQLQ were 2.00, 1.06, and 0.94, respectively, with large effect sizes for NOSE (1.06) and RQLQ (0.94) and a small effect size for TNSS (0.27) (Table II) (Fig 1).

**Table II:** Outcome measures after 5 wk of treatment (n = 35)

Outcome measures	Pretreatment, mean ± SD	Posttreatment, mean ± SD	Mean difference	Effect size (Cohen’s *d*)	*P* value
TNSS	1.971 ± 2.514	1.000 ± 2.776	2.000	0.275	.009∗
NOSE	41.286 ± 23.211	17.857 ± 22.139	23.429	1.061	<.001∗
RQLQ	1.292 ± 0.751	0.587 ± 0.569	0.705	0.943	<.001∗

∗ *P* < .05.

Subdomain analysis of the RQLQ revealed significant reductions in scores for activity (*P* = .003; Cohen’s *d* = 0.572), sleep (*P* < .001; Cohen’s *d* = 0.814), non–hay fever symptoms (*P* < .001; Cohen’s *d* = 0.742), practical problems (*P* < .001; Cohen’s *d* = 0.718), nasal symptoms (*P* = .004; Cohen’s *d* = 0.489), and emotional well-being (*P* < .001; Cohen's *d* = 0.763). The eye symptoms subdomain, however, did not show a statistically significant change (*P* = .340; Cohen’s *d* = 0.187) (Table III) (Fig 1). Adverse events such as exacerbation of symptoms or other discomfort were checked. But no adverse events were observed during the course of intervention.

**Table III:** Subdomains of RQLQ after 5 wk of treatment (n = 35)

Subdomains	Pretreatment, mean ± SD	Posttreatment, mean ± SD	Mean difference	Effect size (Cohen *d*)	*P* value
Activity	0.665 ± 1.037	0.132 ± 0.269	1.180	0.572	.003∗
Sleep	1.750 ± 1.730	0.589 ± 0.960	1.835	0.814	<.001∗
Non–hay fever symptoms	1.796 ± 1.359	0.825 ± 0.960	1.070	0.742	<.001∗
Practical problems	1.45 ± 1.172	0.390 ± 0.879	1.080	0.718	<.001∗
Nasal symptoms	1.364 ±1.106	0.650 ± 1.249	1.000	0.489	.004∗
Eye symptoms	0.436 ± 0.761	0.357 ± 0.731	0.250	0.187	.340
Emotional well-being	1.547 ± 1.502	0.797 ± 1.034	1.000	0.763	<.001∗

∗ *P* < .05.

## Discussion

The study aimed to assess the feasibility, acceptability, safety, and preliminary effects of a nasal rehabilitation in patients with CAR. The patients showed high adherence (92.5%) for the 5 weeks of intervention. The techniques were easy to understand and comfortable to perform for the patients. Furthermore, the therapist reported that the intervention was acceptable by the patients and manageable to apply in the clinical practice. No adverse events were reported during the course of intervention. Overall, the primary findings of the study suggest that the intervention is feasible and safe for patients with CAR.

The TNSS showed significant improvement at the end of 5 weeks, with a mean change of 2 points. It exceeded the established minimal clinically important difference (MCID) of 0.55 points, indicating a clinically meaningful improvement.^25^ Our findings are in line with a previous study by Nair et al^19^ reporting a marked reduction in nasal symptoms, with mean scores decreasing from 5.10 to 3.83 following humming therapy in patients with allergic rhinitis. The comparatively smaller improvement observed in our study may be attributed to the shorter duration of the intervention.

Furthermore, the NOSE score showed notable change, with a mean difference of 23.429 points. However, its MCID values are not yet established. The RQLQ score (0.943 points) exceeded the MCID of 0.5 points, showing the clinical effect.^25^ Our study results matched with those of the previous study done by Courtney et al^20^ in which there was reduction in the mouth breathing with 4 weeks of functional nasal rehabilitation. The physiologic advantages of the components involved in functional nasal rehabilitation such as breath holding, humming, and nose-opening smile play a vital role in this improvement. A study by Hasegawa and Kern^26^ found that breath holding for 30 seconds or longer can decrease nasal resistance in subjects with rhinoscopically normal noses without any history of nasal or paranasal sinus disease. The mechanism by which breath holding reduces nasal resistance involves changes in nasal airflow dynamics and greater nitric oxide production in the nasal cavity.^27^ It also activates the stretch receptor in the nasal area, which is transmitted to the cerebral cortex and helps in changing breathing pattern and reducing mouth breathing.^28^

Moreover, humming increases nasal nitric oxide production of an individual. Nasal nitric oxide is increased 15- to 20-fold by humming compared with quiet exhalation, thereby improving sinus ventilation and reducing inflammation in allergic rhinitis.^29^, ^30^, ^31^ Humming also has a positive impact on psychological outcomes such as anxiety, thereby reducing nasal congestion.^32^ However, the effect of the intervention on these parameters requires further research. Another component of functional nasal rehabilitation involves nose opening during a smile. This causes an activation of specific facial muscles, primarily the levator nasalis and zygomaticus major muscles, which results in strengthening of these muscles.^33^ Evidence suggests that strengthening the levator nasalis and zygomaticus major muscles may improve nasal airflow and reduce mucosal congestion in individuals with CAR. Their activation supports nasal valve dilation and facial drainage, contributing to symptom relief.^34^

This study has certain limitations. First, the present feasibility study lacks a control group. All the patients underwent counseling on allergens avoidance, nasal irrigation, and proper use of corticosteroid spray, which may have contributed to symptom improvement leading to a significant bias. Future trials could include a control group with conventional treatment for understanding the effect of the intervention. Furthermore, we could not use any objective outcome measures such as rhinomanometry, peak nasal inspiratory flow, and acoustic rhinometry.^35^ This was a single-center study, and so the results lack generalizability of the effect of treatment on a broader population, which can be accounted for in future multicenter randomized controlled trials with larger sample sizes. This study evaluated outcomes after 5 weeks, but the persistence of benefit beyond this period is unknown. Future trials can evaluate the long-term effects of nasal rehabilitation.

### Conclusion

Nasal rehabilitation intervention is feasible, acceptable, and safe within the limitations of a small, uncontrolled pilot study in patients with CAR. These patients reported positive preliminary effects with reduced nasal symptoms and enhanced disease-specific quality of life after 5 weeks of nasal rehabilitation.

## Disclosure statement

Disclosure of potential conflict of interest: The authors declare that they have no relevant conflicts of interest.

Data availability statement: The data used in this study are available from the corresponding author.

Ethical statement: Approval was provided by the Institutional Research Committee of Manipal College of Health Professions, Manipal Academy of Higher Education, Manipal, and the ethical committee of Manipal Hospitals, Bengaluru, to conduct this study. Informed consent was taken from all the subjects before their participation in the study.
