# Supplementary material for: Feasibility and efficacy of nasal rehabilitation on nasal symptoms in patients with chronic allergic rhinitis: A pilot study
Source: J Allergy Clin Immunol Glob. 2026 Feb 17;5(3):100674. doi: 10.1016/j.jacig.2026.100674 (PMC12993898; doi:10.1016/j.jacig.2026.100674)
Supplement: Supplementary Methods [file mmc1.docx]

**NASAL REHABILITATION PROTOCOL**

| **Exercise** | **Patient position** | **Procedure** | **Repetitions** |
| --- | --- | --- | --- |
| Breath holding | sitting in a chair in a relaxed manner | Deep inhalation followed by breath hold of 10 seconds and exhale. Gradually, the time of breath holding was increased to 30 second. | Twice a day with 5 repetitions each |
| Humming | sitting in a chair | Bilateral humming - inhale and make a sound hum while exhaling with both the nostril.  Unilateral humming - inhale and make a sound hum while exhaling through unilateral nostril. | Twice a day with 5 repetitions each |
| Nose opening smile | sitting in a chair | Start by sitting up straight, keeping your spine aligned, your head balanced over your shoulder and chin gently tucked in. Place your tongue against the roof of your mouth. Bring your attention to the sensations in your tongue and nose, becoming aware of the airflow during both inhalation and exhalation. As you breathe, use your imagination or memory to recall a pleasant, mouth-watering aroma, or simply enjoy the sensation of air passing through your nose while gently opening your nostrils, smiling, and relaxing your body. To enhance nasal openness, smile broadly, raise your eyebrows, and flare your nostrils slightly as you continue to breathe deeply and calmly  NOS variation- same technique with low slow breathing. | Twice a day with 5 repetitions each |
